# No Quarantine

**Published:** 1893-01-28

**Authors:** 


### NO QUARANTINE.

Dr. Krocker, in an exhaustive article in the "Deutsche Rundschau" on the cholera year, 1892, pours unbounded contempt on the precautions commonly advocated in Germany against the spread of cholera. He shows that the military cordon with which it has been proposed to surround infected towns and districts must be altogether inadequate to the task, since the united armies of Germany proved insufficient for the effectual blockade of Paris. He strongly deprecates also the enforcement of quarantine, on the ground of the dangers incurred by herding numbers of persons together in a time of panic. He argues that the cholera germ, like most other evils, becomes less formidable on more familiar acquaintance. Recent experiments at close quarters with these germs seem to prove that they are capable of being communicated to the system solely through the mouth, thence from the stomach to the intestines, where their real period of activity begins. Until communicated to the mouth the germ subsists in so attenuated a form that neither an infected atmosphere nor actual contact with an infected person need be dreaded if food and drink, with all vessels and hands used in their preparation and consumption, be properly guarded or purified. Dr. Krocker urges upon the Government the enforcement of a universal and rigid revision system. The control of the water supply takes a leading place in his proposed measures, which are similar in almost all points to those already urged by the English authorities. Finally, Dr. Krocker would allow free circulation of the people during cholera time, but all travellers from infected districts under his revision system would be obliged to undergo a prompt disinfection of themselves and their effects. This, we believe, would be eagerly welcomed by travellers if carried out with reasonable attention to their comfort, as a substitute for the delays and annoyance of quarantine.

